# Berberine ameliorates depression-like behaviors in mice via inhibiting NLRP3 inflammasome-mediated neuroinflammation and preventing neuroplasticity disruption

**DOI:** 10.1186/s12974-023-02744-7

**Published:** 2023-03-01

**Authors:** Zongshi Qin, Dong-Dong Shi, Wenqi Li, Dan Cheng, Ying-Dan Zhang, Sen Zhang, Bun Tsoi, Jia Zhao, Zhen Wang, Zhang-Jin Zhang

**Affiliations:** 1 Peking University Clinical Research Institute, Peking University, Beijing, China; 2 School of Chinese Medicine, LKS Faculty of Medicine, The University of Hong Kong, Hong Kong SAR, China; 3 Shanghai Mental Health Center, Shanghai Jiao Tong University School of Medicine, Shanghai, China; 4 Department of Food Science and Nutrition, The Hong Kong Polytechnic University, Hong Kong SAR, China

**Keywords:** Depression, Berberine, NLRP3 inflammasome, Neuroinflammation, Synaptic plasticity

## Abstract

**Objectives:**

Neuroinflammation has been suggested that affects the processing of depression. There is renewed interest in berberine owing to its anti-inflammatory effects. Herein, we investigated whether berberine attenuate depressive-like behaviors via inhibiting NLRP3 inflammasome activation in mice model of depression.

**Methods:**

Adult male C57BL/6N mice were administrated corticosterone (CORT, 20 mg/kg/day) for 35 days. Two doses (100 mg/kg/day and 200 mg/kg/day) of berberine were orally administrated from day 7 until day 35. Behavioral tests were performed to measure the depression-like behaviors alterations. Differentially expressed gene analysis was performed for RNA-sequencing data in the prefrontal cortex. NLRP3 inflammasome was measured by quantitative reverse transcription polymerase chain reaction, western blotting, and immunofluorescence labeling. The neuroplasticity and synaptic function were measured by immunofluorescence labeling, Golgi–Cox staining, transmission electron microscope, and whole-cell patch-clamp recordings.

**Results:**

The results of behavioral tests demonstrated that berberine attenuated the depression-like behaviors induced by CORT. RNA-sequencing identified that NLRP3 was markedly upregulated after long-term CORT exposure. Berberine reversed the concentrations of peripheral and brain cytokines, NLRP3 inflammasome elicited by CORT in the prefrontal cortex and hippocampus were decreased by berberine. In addition, the lower frequency of neuronal excitation as well as the dendritic spine reduction were reversed by berberine treatment. Together, berberine increases hippocampal adult neurogenesis and synaptic plasticity induced by CORT.

**Conclusion:**

The anti-depressants effects of berberine were accompanied by reduced the neuroinflammatory response via inhibiting the activation of NLRP3 inflammasome and rescued the neuronal deterioration via suppression of impairments in synaptic plasticity and neurogenesis.

**Supplementary Information:**

The online version contains supplementary material available at 10.1186/s12974-023-02744-7.

## Introduction

Depression has been identified as a high incidence and severe psychiatric disease [1]. The health burden of depression by using disability-adjusted life-years (DALYs) estimation accounted for 1.85% of all DALYs worldwide, which increased 61.1% from 1990 to 2019 [2]. It is known to significantly increase the risk of suicide for all ages, especially in adolescents. Although the understanding of the pathology of depression has developed considerably. Currently, no single mechanism can satisfactorily explain the pathophysiology of depression [3, 4]. Studies have focused on many components of brain including prefrontal cortex (PFC), hippocampus, amygdala, ventral tegmental area (VTA), and nucleus accumbens (NAc), leading to the theories of depression as well as antidepressant response that have been involved in the molecular and cellular signaling mechanisms that mediate synaptic plasticity, contributing to a broader neuroplasticity hypothesis of depression [5, 6]. To date, increasing evidence indicated that overexpressed peripheral inflammatory responses could injure the integrity of the blood–brain-barrier (BBB) and result in neuroinflammation in the brain [7]. Consequently, the neuroinflammation-mediated neuroplasticity and neurogenesis defects might be a vital process under the mechanism in neuropsychiatric conditions, including depression [8]. It has been observed that an excess in peripheral acute phase proteins and proinflammatory cytokines production in depression patients, which have been identified to be linked with emotional alterations and severity of psychiatric symptoms [9, 10]. Besides, the remission of patients is often occurring after normalization of the inflammatory response, whereas a failure to remission is accompanied by the persistently elevated inflammatory response. This information promotes the hypothesis that the emotional alterations in depression patients might be attributed by an anomalous link between the central nervous system (CNS) and the innate immune response. Moreover, the pooled data from meta-analysis also supported that several anti-inflammatories have significant antidepressant effects [11].

The interactions between the immune system and the CNS are not only involved in shaping behavior, but also in responding to therapeutics [12]. NLRP3 (NLR family, pyrin domain containing 3) inflammasome complex is an intracellular multiprotein complex responsible for several innate immune processes associated with infection, inflammation, and autoimmunity [13]. As a component of the innate immune system that functions as a pattern recognition receptor that recognizes pathogen-associated molecular patterns (PAMPs) and danger-associated molecular patterns (DAMPs), NLRP3 appears to bridge the gap between immune activation and metabolic danger signals or stress exposure, which might be key factors in the pathogenesis of depression [14, 15]. Stimulated by NLRP3, the over-released pro-inflammatory cytokine IL-1β can cross the BBB and alter synaptic plasticity by directly acting on neurons or stimulating the microglia activation [16–18]. In addition, the hypothalamus–pituitary–adrenal (HPA) axis could be stimulated by cytokines and result in glucocorticoids overproduction, exacerbating the stress response [19, 20]. On the other hand, the neurotoxic effects of neuroinflammation consequently contribute to the synaptic remodeling, suggesting that neural plasticity also plays a vital role in the pathophysiology of depression and antidepressant function [21]. High levels of inflammatory molecules have been reported to decrease a wide range of neural plasticity markers such as synaptic transmission, membrane excitability, plasticity in pyramidal neurons, as well as neurogenesis. NLRP3 matured IL-1β plays functional roles in the mechanisms of synaptic plasticity and cognitive functions. In the depression mice, the spine density and critical morphologies were significantly decreased, especially in the specific brain regions related to depression, such as the prefrontal cortex and hippocampus.

Berberine is a natural isoquinoline alkaloid and there is renewed interest in berberine of its potential role in neurodegenerative and neuropsychiatric disorders because of its effect on neuroinflammation, hormonal regulation, and neurotransmitters [22–24]. In this study, we investigated the differentially expressed genes in corticosterone induced depression mice model (CORT) using a high-throughput microarray. The theory that disruption of neurotrophic factors and synaptic connectivity in the PFC and hippocampus is related to neuroplasticity mechanisms is one of the leading neuroplasticity hypotheses of depression [25]. We found that NLRP3 showed significantly differential expression within the PFC of CORT-induced mice model versus wildtype mice controls and berberine-treated mice. Complementing these findings, the CORT mice result in neuroplasticity deficits and neurogenesis injury and induced depression-like behaviors. Accordingly, these results provide insights into mechanisms involving the functional regulation of corticosterone in depression and specifically, identify berberine as a potential therapy for depression.

## Materials and methods

### Animal models and drug treatment

**Fig. 1:**
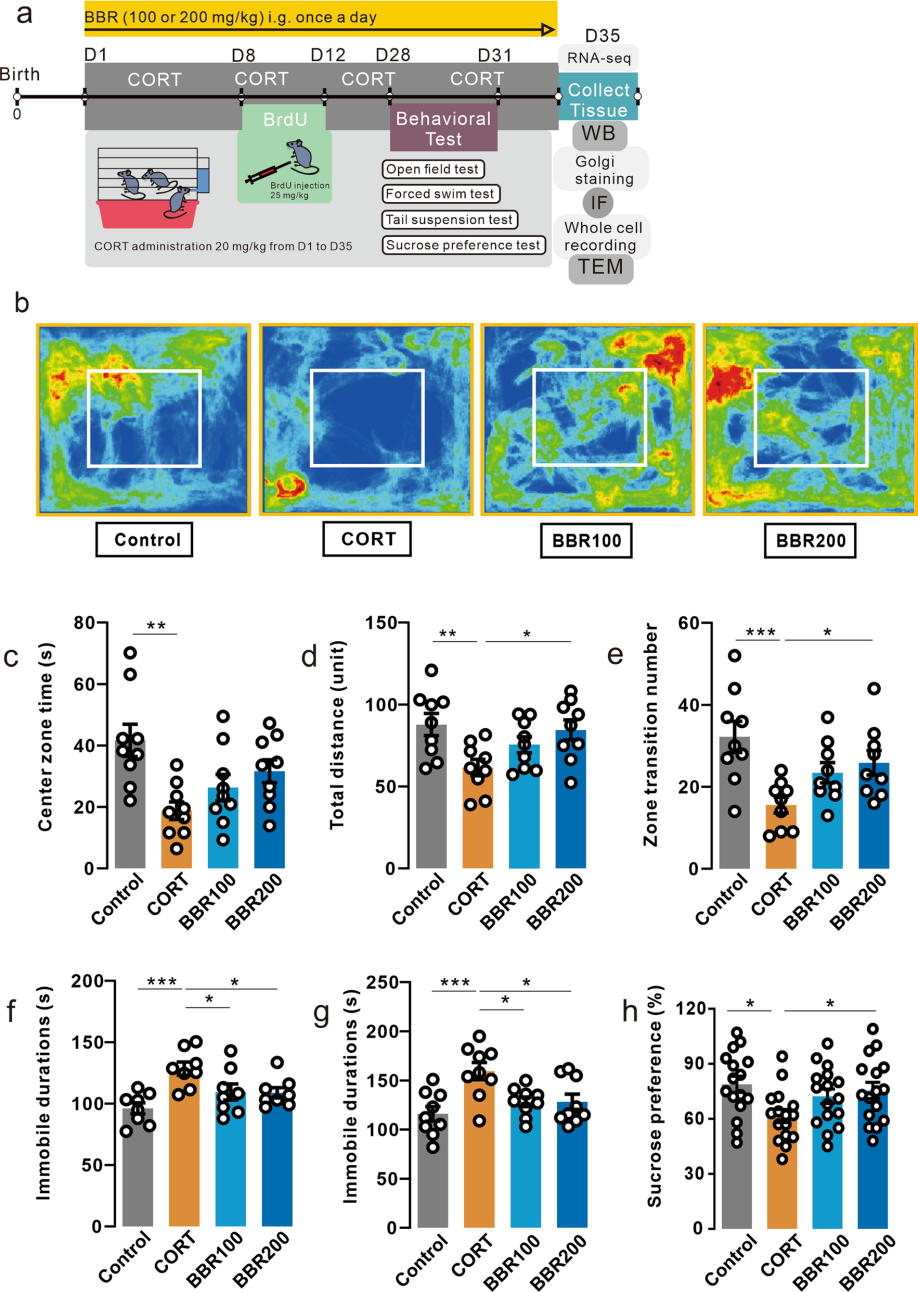
Berberine prevents CORT-induced behavioral changes.** a** Schematic of experiment paradigm. CORT was consecutively administered from day1 to day35. From day7, 100 mg/kg/day or 200 mg/kg/day of berberine were given to mice in BBR100 or BBR200 groups via daily intragastrical administration, respectively. Mice in control and CORT groups received solution without berberine. Mice were given BrdU as daily intraperitoneal injections from day8 to day12. From day28 to day31, a series of behavioral tests were conducted for mice, CORT and berberine administration were sustained during behavioral tests until to the end of the experiment (day35). **b** Heatmap of mice exploration during the open field test. **c** Duration in central zone in the open field test. CORT mice spent less time in the central zone than the control mice (One-way ANOVA, *F* (3, 32) = 5.448, *P* = 0.0038; Dunnett’s multiple comparisons test, CORT vs. Control, *P* = 0.0012, *n* = 9 in each group). **d** Total distance in the open field test. CORT mice moved less in the field than the control and high-dose berberine mice (One-way ANOVA, *F* (3, 32) = 4.203, *P* = 0.0129; Dunnett’s multiple comparisons test, CORT vs. Control, *P* = 0.0077, CORT vs. BBR200, *P* = 0.0212, *n* = 9 in each group). **e** Zone transition number in the open field test. CORT administration decreased the number of zone transitions in mice in the open field test compared to the control and high-dose berberine mice (One-way ANOVA, *F* (3, 32) = 5.711, *P* = 0.0030; Dunnett’s multiple comparisons test, CORT vs. Control, *P* = 0.0008, CORT vs. BBR200, *P* = 0.0429, *n* = 9 in each group). **f** Immobile duration in the tail suspension test. CORT mice spent more time in immobile duration (One-way ANOVA, *F* (3, 28) = 6.435, *P* = 0.0019; Dunnett’s multiple comparisons test, CORT vs. Control, *P* = 0.0005, CORT vs. BBR100, *P* = 0.0425, CORT vs. BBR200, *P* = 0.0345, *n* = 8 in each group). **g** Immobile duration in the forced swim test. CORT mice spent more time in immobile duration (One-way ANOVA, *F* (3, 32) = 6.316, *P* = 0.0017; Dunnett’s multiple comparisons test, CORT vs. Control, *P* = 0.0007, CORT vs. BBR100, *P* = 0.0145, CORT vs. BBR200, *P* = 0.0141, *n* = 9 in each group). **h** Sucrose consumption in the sucrose preference test. The consumption of sucrose in CORT mice was significantly lower than in the mice in control and high-dose berberine groups (One-way ANOVA, *F* (3, 32) = 4.638, *P* = 0.0084; Dunnett’s multiple comparisons test, CORT vs. Control, *P* = 0.0070, CORT vs. BBR200, *P* = 0.0090, *n* = 16 in each group). Bar graphs show the mean ± SEM; *P < 0.05, **P < 0.01, ***P < 0.001. *CORT* corticosterone, *BBR* berbberine, *ANOVA* analysis of variance

Adult male C57BL/6N mice (age 8–10 weeks) were obtained from the Centre for Comparative Medicine Research (CCMR), the University of Hong Kong. All mice were raised in the experimental holding areas (12 h light/dark cycle at 18–22 °C, with lights on at 8:00 A.M., ad libitum access to dry food pellets and water) following the ethics roles of the HKU (CULATR No. 5582-20) in the CCMR. Body weight was daily recorded during the entire experimental period. The mice were divided into 4 groups (*n* = 18 for each group), including saline-treated (control), corticosterone (20 mg/kg/day) treated (CORT), CORT + berberine (100 mg/kg/day) treated (BBR100), and CORT + berberine (200 mg/kg/day) treated (BBR200). CORT was consecutively administered from day1 to day35. From day7, 100 mg/kg/day or 200 mg/kg/day of berberine were given to mice in BBR100 or BBR200 groups via daily intragastrical administration, respectively. Mice in control and CORT groups received solution without berberine. Mice were given BrdU as daily intraperitoneal injections from day 8 to day 12. From day 28 to day 31, a series of behavioral tests were conducted for mice, CORT and berberine administration were sustained during behavioral tests until to the end of the experiment (day35). The dose of CORT was selected in accordance with a previous study that successfully induced depression in mice from the same laboratory [26]. CORT and berberine were dissolved in a 0.5% aqueous solution of sodium carboxymethyl cellulose and administered via oral gavage. The solutions were freshly prepared before daily use. Figure 1a illustrates the experimental design timeline with the time of assays and manipulations.

### Behavioral tests

#### Open field test (OFT)

For assessment of locomotion and anxiety-like behavior in mice. The OFT was performed in a white plastic apparatus (50 height × 50 widths × 40 cm depth) with a 30 × 30 cm central zone. The total distance and velocity moved and the frequency of transfer between the central and surrounding zones was recorded over a 10-min test period. The 95% ethanol was sprayed to clean the apparatus between each test to avoid odor and waste left by the last mouse. We performed the test for all mice on the first day before the experiment. SMART video tracking software (V.3.0, Panlab, USA) was used to record and analyzed data.

#### Tail suspension test (TST)

The TST was performed in a white plastic chamber (55 height × 10 widths × 10 cm depth). Each mouse was suspended from its tail tip with adhesive tape in a head-down position and lasted for 6 min. Immobility time is defined as the cessation of any movements of limbs and the trunk. To avoid the bias affected by the stress response, each mouse was adapted for 2 min after being suspended, only the remaining 4 min was recorded and analyzed. SMART video tracking software (V.3.0, Panlab, USA) was used to record and analyzed data.

#### Forced swimming test (FST)

To evaluate a depressive-like behavioral state, the FST was performed in a clear polycarbonate cylinder (30 height × 20 cm diameter). Each mouse was forced to swim in each cylinder filled with water (23 to 25 °C) for 6 min and videotaped. Immobility time is defined as the absence of all movements except the motions required to keep the mice's heads above the surface of the water. To avoid the bias affected by the stress response, each mouse was adapted for 2 min in the water, only the remaining 4 min was recorded and analyzed.

#### Sucrose preference test (SPT)

SPT was performed to assess the anhedonia, a core symptom of depression. First, mice were adaptively exposed to two bottles containing 1% sucrose solution (w/v) with ad libitum access for 24 h in groups of 5 per cage. Then, one bottle of 1% sucrose solution and another bottle of tap water were administrated for 24 h. On the last day, the position of the two bottles was switched for 24 h to avoid side preference. At the end of the adaptation period, all mice were deprived of food and water for 12 h before the test. After that, SPT was conducted in an individual mouse housed in a cage with free access to two respective bottles containing 1% sucrose solution and tap water for 2 h. To prevent side preferences in drinking behavior, the position of the two bottles was switched in the middle of the testing. Water and sucrose consumption was measured as changes in the weight of fluid consumed. The sucrose preference was calculated from the following formula was the sucrose preference (%) = the sucrose consumption (g)/[the sucrose consumption(g) + the water consumption (g)] × 100%.

### RNA isolation, qPCR and RNA-sequencing

Total RNA was isolated from dissected prefrontal cortex using QIAzol lysis reagent and purified using a miRNAeasy mini kit (Qiagen). cDNA was acquired from total RNA using a high-capacity cDNA Reverse Transcription Kit (Life Technologies). qPCR was performed using the Fast Start Universal SYBR Green Master kit (Takara, Japan) using CFX96 Real-Time System (Bio-Rad, USA). Each reaction was performed in triplicates. The relative quantification was determined by the ΔΔCT method. The values were normalized to those of β-actin mRNA in the same cDNA samples. Additional file 1: Table S1 summarizes the information on primers for qPCR. The details for library preparation and transcriptome sequencing are shown in the supplementary information.

### Brain slice preparation and electrophysiology recording

After the end of behavioral tests, mice were anesthetized with a combined anesthetic (ketamine 100 mg/kg and xylazine 10 mg/kg i.p.) then perfused with chilled dissection buffer (110 mM choline chloride, 25 mM NaHCO_3_, 1.25 mM NaH_2_PO_4_, 2.5 mM KCl, 0.5 mM CaCl_2_, 7 mM MgCl_2_, 11.6 mM ascorbic acid, 3.1 mM pyruvic acid, and 25 mM d-glucose). The brains were immediately transferred into ice-cold oxygenated (95% O_2_ and 5% CO_2_) dissection buffer and coronal mPFC slices (300 µm) were cut using a vibratome (VT1000s, Leica, Germany). Then, slices were incubated in oxygenated (95% O_2_/5% CO_2_) artificial cerebrospinal fluid solution (aCSF; 118 mM NaCl, 2.5 mM KCl, 26.5 mM NaHCO_3_, 1 mM NaH_2_PO_4_, 1 mM MgCl_2_, 2 mM CaCl_2_, and 20 mM D-glucose) at room temperature for 60 min to recover from the mechanical shock of slicing.

The brain slices were soaked in the running artificial cerebrospinal fluid solution at 5 mL/min flow rate in the holding chamber of the electrophysiology recording platform. The glass recording pipettes were made by a pipette puller (P-97, Sutter Instrument, USA). The cells were located by a fine control micromanipulator under an optical microscope (Zeiss, Germany). The resistance ranged between 2 and 5 MΩ following fire polishing to enhance seal quality. For current-clamp recordings, the intracellular solution contained (in mM) 130 K gluconates, 5 KCL, 10 HEPES, 2.5 MgCl_2_, 4 Na_2_ATP, 0.4 Na_3_GTP, 10 Na phosphocreatine, 0.6 EGTA. For voltage-clamp recordings, the intracellular solution contained (in mM) 115 CsMeCO3, 20 CsCl, 10 HEPES, 2.5 MgCl2, 4 Na2ATP, 0.4 NaGTP, 10 Na phosphocreatine, and 0.6 EGTA. Current clamp recordings were filtered at 2.5 kHz and sampled at 5 kHz. Voltage clamp recordings were filtered at 2.5 kHz and sampled at 10 kHz. The sequential currents from − 50 to 400 pA in a 50 pA step for 500 ms were injected. The currents were injected every 60 s in the current clamp. The electrophysiology recording was performed using a patch-clamp amplifier (EPC10USB, HEKA, Germany), and the spontaneous or miniature excitatory events were analyzed with Mini Analysis Program (v.6.0.3, Synaptosoft Inc., USA).

### Enzyme-linked immunosorbent assay (ELISA)

Serum concentrations of cytokines including IL-1β (KE10003, Proteintech), IL-6 (KE00007, Proteintech), IL-10 (KE00170, Proteintech), and TNF-α (KE10002, Proteintech) were measured using the quantitative ELISA kits. All measurements were performed in duplicate. The absorbance of each well for reactions was detected at 450 nm using the CLARIOstar Plus Microplate Reader (BMG LABTECH, Germany). The cytokine concentrations were determined by the standard curve of each cytokine.

### Western blotting

To determine the expression of the protein, the hippocampus and prefrontal cortex tissue was extracted by RIPA buffer (Sigma-Aldrich, USA) containing 1% protease inhibitor cocktail (MCE, USA). The protein concentrations were detected by Bradford protein assay using Coomassie brilliant blue G-250 (Bio-red Laboratories Inv., USA). Proteins were separated via 10% or 12% SDS–PAGE gels according to the different protein sizes and transferred to PVDF membranes (0.45 μm, Bio-red Laboratories Inv., USA). The blots were subsequently blocked for 1 h with 5% BSA at room temperature. After being incubated with primary antibodies at 4 ℃ overnight, the membranes were incubated with fluorescence-conjugated secondary antibodies for 1 h at room temperature. Proteins were detected using an ECL kit (GE Healthcare, UK) and quantified using the Image Lab software (v.5.2.1, Bio-Rad, USA). The details of antibody information are shown in Additional file 1: Table S2.

### Immunofluorescence labeling

The BrdU was injected for 5 days continuously from day 8 to day 12, mice were sacrificed by cardiac perfusion with 4% paraformaldehyde. Brains were collected postfixed in 4% paraformaldehyde at 4 ℃ overnight and then dehydrated in 30% sucrose PBS solution for 2 days at 4 ℃ cold room. Coronal brain sections (25 μm) were prepared with a freezing microtome (Leica Inc., Germany). For immunofluorescence study, slides were processed antigen retrieved with citrate acid buffer with microwave oven for 30 min. Next, slides were blocked by BSA for 60 min at room temperature and then incubated at 4 ℃ overnight in the primary antibodies. After washing by PBST, the slides were incubated with fluorescent-dye-conjugated secondary antibodies (DyLight 594-conjugate donkey anti-goat, 1:200, Abcam; DyLight 488-conjugate donkey anti-rabbit, 1:200, Abcam; DyLight 594-conjugate donkey anti-rabbit, 1:200, Abcam) in dark for 1 h at room temperature. Before mounting, the slides were stained with DAPI to stain the nucleus for 10 min. Images were captured using a confocal microscope (LSM880, Zeiss, Germany). Analyses of pictures were performed using the ImageJ software (v.1.53c, NIH, USA). The details of antibody information are shown in Additional file 1: Table S3.

### Golgi-Cox staining

Golgi-Cox staining was performed by using the commercial staining kit (Hito Golgi-Cox OptimStain Kit, Hitobiotec Corp, USA) to characterize potential changes in the density and feature of neuronal dendritic spines. The whole brain was soaked in the staining solution at room temperature for 14 days to avoid light according to the instruction manual. The tissues were prepared as paraffin sections into 60 μm. Images were captured using a confocal microscope (LSM880, Zeiss, Germany). Three-D reconstruction was performed by using Imaris software (v.9.0.1, Bitplane AG, Switzerland) to detect the categories of dendrite spine, the cells with straight terminal branches that had a clear resolution of spines and were longer than 10 μm were selected for dendritic spines counting and analysis.

### Transmission electron microscope (TEM)

After perfusion with 4% paraformaldehyde, the hippocampus of mice was manually cut into 1 mm^3^ tissue and immediately fixed in 2.5% glutaraldehyde in 0.1 M phosphate buffer overnight at 4 °C. The tissues were transferred into 4% osmium tetroxide with 3% potassium ferrocyanide in 0.1 M cacodylate buffer for 1 h at 4 °C and avoid light then embedded in Epon 812 after dehydration. Samples were sectioned into fine sections (0.4 μm) using an ultramicrotome (Ultracut UCT, Leica, USA) and moved to copper grids. After staining with 2% aqueous uranyl acetate and followed by Reynold’s lead citrate, the images were captured by using a transmission electron microscopy (CM100, Philips, Germany) at 100 kV attached with a charge-coupled device camera (SIS, Olympus, Japan).

### Statistical analysis

Data are expressed as mean ± standard error of the mean with two-sided *p* < 0.05 considered significant. PROC POWER of SAS (v.9.4, SAS Institute Inc, USA) was used to determine the proper sample size for animal experiments. One-way or two-way analysis of variance (ANOVA) was appropriately used for comparisons among multiple groups in terms of behavioral tests, qPCR, western blot, ELISA, and immunofluorescence, followed by Dunnett’s or Tukey’s post hoc multiple comparisons adjustment. Data analyses were performed using GraphPad Prism software (v.9.0.0, GraphPad Software, USA). To detect correlation between behavioral parameters as dependent variables and NLRP3 inflammasome as independent variables, the Spearman’s correlation coefficient was calculated and the correlation matrix showing correlation coefficients between variables were summarized. The quantification analysis of immunofluorescence staining was calculated according to cell density analysis. One out of four 25 μm thick sections were analyzed thus representing the entire rostrocaudal extent of different brain regions at × 20 magnification. For analysis of NLRP3 + and Iba-1 + cells in PFC and hippocampus, the positive cells were counted and compared between groups. For analysis of PSD95 + , SYN + , BrdU + and DCX + cells in hippocampus, the positive cells were separately counted. Cells were counted using the plugin “Manual counting” (https://icy.bioimageanalysis.org/plugin/manual-counting/). The details for bioinformatic analysis for RNA-seq data are shown in the supplementary information.

## Results

### Berberine attenuates the emotional dysfunctions in CORT-induced depression mice

Two doses of berberine (100 mg/kg/day and 200 mg/kg/day) were orally administrated daily from the middle of the CORT modeling until the end of the behavioral tests (Fig. 1a). Long-term high-dose CORT administration significantly decreased the body weight of mice, and a high dose of berberine reversed the negative effects of CORT on the body weight (Additional file 1: Fig. S1). CORT markable decreases the center zone time duration, total distance as well as zone transition number in OFT in mice (Fig. 1b–e). The high dosage (200 mg/kg/day) of berberine significantly reversed CORT-induced behavioral alterations (Fig. 1c–e). In the TST, CORT significantly increased immobile duration compared to other groups (Fig. 1f) and the same pattern was observed in the FST (Fig. 1g). In the SPT, the results showed a significant effect of CORT on the consumption of sucrose solution, the high dosage (200 mg/kg/day) of berberine reversed CORT-induced behavioral alterations (Fig. 1h). Collectively, these results suggest that berberine treatment especially in high doses (200 mg/kg) could attenuate the behavioral alterations associated with depression induced by CORT.

### Berberine reverses the peripheral and brain cytokine levels

**Fig. 2:**
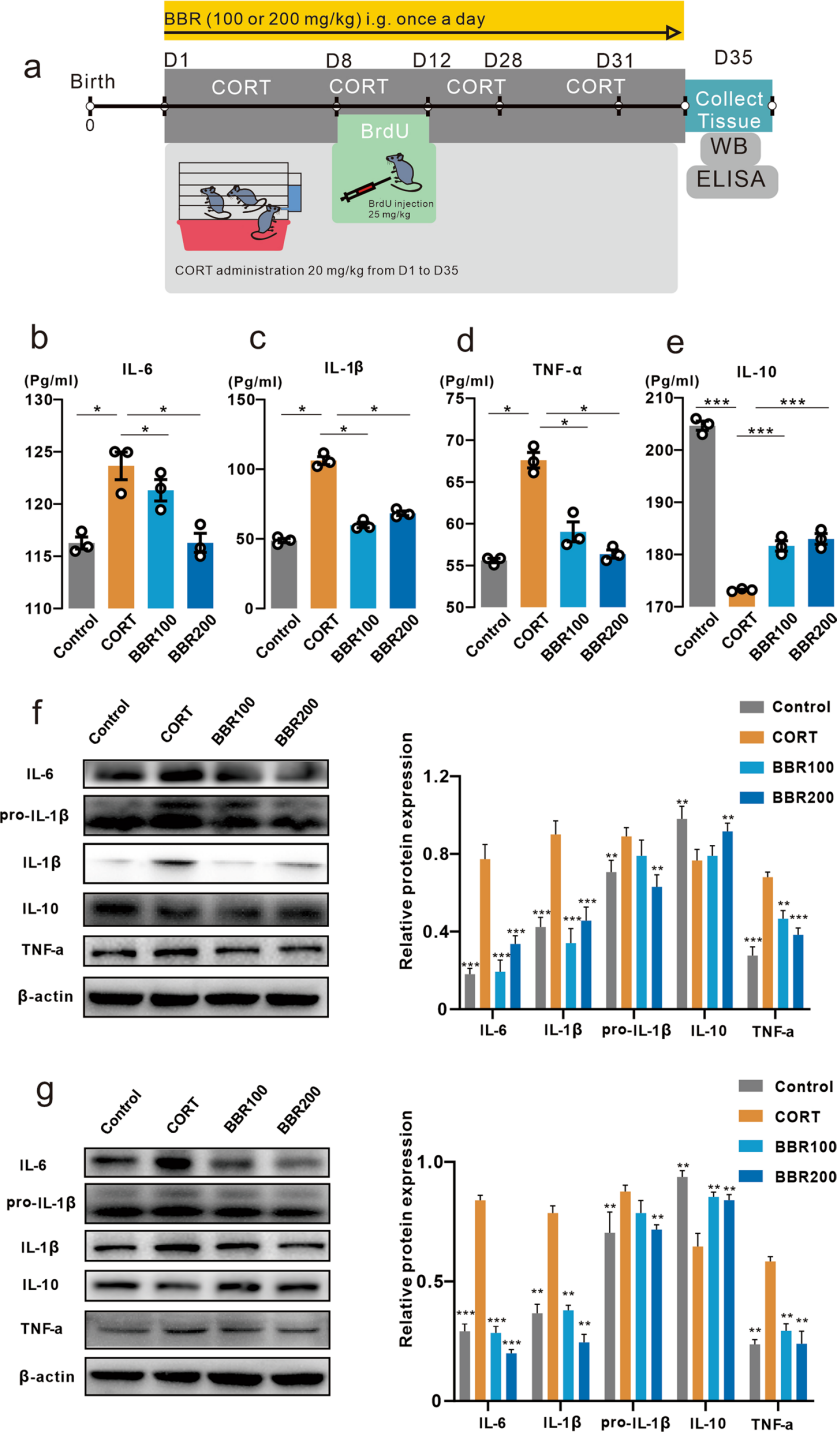
Effects of berberine on serum cytokines and neuroinflammatory responses in the prefrontal cortex and hippocampus.** a** Timing of CORT administration, behavioral tests, and western blotting and ELISA assay. **b** Effects of berberine on serum cytokine level of IL-6. The CORT significantly increased the IL-6 concentration compared to the mice in control and high-dose berberine groups (One-way ANOVA, *F* (3, 8) = 13.80, *P* = 0.0016; Dunnett’s multiple comparisons test, CORT vs. Control, *P* = 0.0020, CORT vs. BBR200, *P* = 0.0021, *n* = 3 in each group). **c** Effects of BBR on serum cytokine level of IL-1β. The CORT significantly increased the IL-1β concentration (One-way ANOVA, *F* (3, 8) = 147.1, *P* < 0.0001; Dunnett’s multiple comparisons test, all comparisons *P* < 0.0001, *n* = 3 in each group). **d** Effects of berberine on serum cytokine level of TNF-α. The CORT significantly increased the TNF-α concentration (One-way ANOVA, *F* (3, 8) = 46.96, *P* < 0.0001; Dunnett’s multiple comparisons test, all comparisons *P* < 0.0001, *n* = 3 in each group). **e** Effects of berberine on serum cytokine level of IL-10. The CORT significantly decreased the IL-10 concentration (One-way ANOVA, *F* (3, 8) = 253.1, *P* < 0.0001; Dunnett’s multiple comparisons test, all comparisons *P* < 0.0001, *n* = 3 in each group). **f** The effects of berberine on cytokine level in prefrontal cortex measured by western blot. The CORT mice showed the significant different in four cytokines (Two-way ANOVA, row factor *F* (3, 41) = 26.78, *P* < 0.0001, column factor *F* (3, 41) = 13.61, *P* < 0.0001; Dunnett’s multiple comparisons test, CORT vs. Control, *P* < 0.0001, CORT vs. BBR100, *P* < 0.0001, CORT vs. BBR200, *P* = 0.0003, *n* = 3 in each group). **g** The effects of berberine on cytokine level in hippocampus measured by western blot. The CORT mice showed the significant different in four cytokines (Two-way ANOVA, row factor *F* (3, 41) = 18.07, *P* < 0.0001, column factor *F* (3, 41) = 8.34, *P* = 0.0002; Dunnett’s multiple comparisons test, CORT vs. Control, *P* = 0.0007, CORT vs. BBR100, *P* = 0.0010, CORT vs. BBR200, *P* = 0.0003, *n* = 3 in each group). Bar graphs show the mean ± SEM; *P < 0.05, **P < 0.01, ***P < 0.001. *CORT* corticosterone, *BBR* berberine, *ANOVA* analysis of variance, *ELISA* enzyme-linked immunosorbent assay

The cytokines of TNF-α, IL-1β, IL-6, and IL-10 were evaluated in the blood, prefrontal cortex (PFC), and hippocampus using ELISA and western blotting assay (Fig. 2a). One-way ANOVA analysis showed a significant effect of CORT on both peripheral, PFC, as well as hippocampus. Berberine effectively prevented the CORT-induced increase in pro-inflammatory cytokines, while increasing the anti-inflammatory cytokine IL-10 reduced by CORT (Fig. 2b–e). The results of western blotting in the PFC and hippocampus showed a similar pattern as ELISA that CORT treatment significantly enhanced proinflammatory cytokines levels including TNF-α, IL-1β, and IL-6. Two doses of berberine markedly reversed the levels of cytokines in the PFC and hippocampus (Fig. 2f, g).

### Bioinformatic analysis of RNA-seq and qPCR analysis identified NLRP3 as a hub gene in CORT mice

**Fig. 3:**
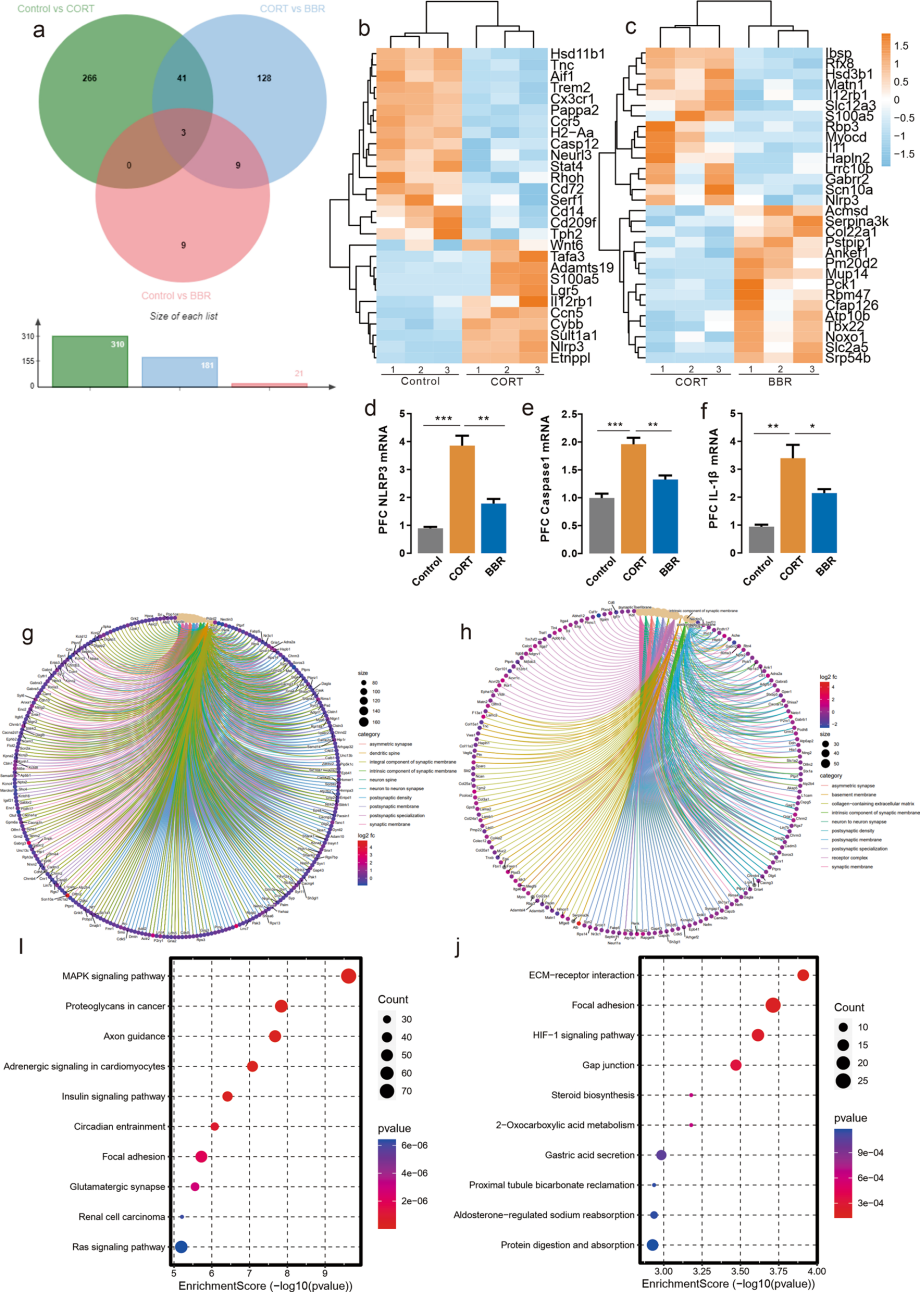
Analysis of RNA-seq. **a** Representation of the differentially expressed genes represented by the Venn diagram. **b** Representation of the heatmap represented top differentially expressed genes between CON and CORT groups, *n* = 3 in each group. **c** Representation of the heatmap represented top differentially expressed genes between CORT and BBR groups, *n* = 3 in each group. **d** Results of qPCR showed that NLRP3 expression in PFC in CORT was higher than in CON and BBR mice (One-way ANOVA, *F* (2, 6) = 44.11, *P* = 0.0003; Dunnett’s multiple comparisons test, CORT vs. Control, *P* = 0.0002, CORT vs. BBR, *P* = 0.0012, *n* = 3 in each group). **e** Results of qPCR showed that caspase-1 expression in PFC in CORT was higher than in CON and BBR mice (One-way ANOVA, *F* (2, 6) = 28.61, *P* = 0.0009; Dunnett’s multiple comparisons test, CORT vs. Control, *P* = 0.0006, CORT vs. BBR, *P* = 0.0049, *n* = 3 in each group). **f** Results of qPCR showed that IL-1beta expression in PFC in CORT was higher than in CON and BBR mice (One-way ANOVA, *F* (2, 6) = 17.86, *P* = 0.0030; Dunnett’s multiple comparisons test, CORT vs. Control, *P* = 0.0018, CORT vs. BBR, *P* = 0.0397, *n* = 3 in each group). **g** Representation of the top associated gene ontology terms between CON and CORT groups. **h** Representation of the top associated gene ontology terms between CORT and BBR group. **i** Representation of the top KEGG enrichment pathways between CON and CORT groups. **j** Representation of the top KEGG enrichment pathways between CORT and BBR group. *CON* control, *CORT* corticosterone, *BBR* berberine, *ANOVA* analysis of variance, *NLRP3* NOD-like receptor thermal protein domain associated protein 3, *qPCR* quantitative reverse transcription polymerase chain reaction, *PFC* prefrontal cortex

To explore the transcriptome-wide alterations after long-term CORT exposure in the mice brain, differentially expressed gene (DEG) of RNA sequencing data from the prefrontal cortex was analyzed to identify a hub gene. Additional file 1: Table S4 shows the quality control of mapped reads for each RNA-seq sample. Long-term administration of high concentration CORT significantly altered the expression of 310 genes compared with the control group, and berberine (200 mg/kg/day) altered the expression of 128 genes compared with the CORT group (Fig. 3a). Forty-four genes were altered at criteria between the control group and CORT group, and the heatmap of DEG lists revealed that genes were altered in the opposite direction. DEG analysis identified NLRP3, as one of the hub genes among those altered in both comparisons of control vs. CORT and CORT vs. berberine. Long-term CORT-administration potentially increased the expression of NLRP3 inflammasome in the PFC compared to normal raised mice and the berberine treated mice (Fig. 3b, c). The mRNA expression alteration of NLRP3, caspase-1 and IL-1β in the PFC was confirmed by qPCR assay (Fig. 3d–f). DEGs were enriched for several relevant gene ontology (GO) terms. GO cellular component analysis indicated that CORT exhibited significant differences in genes involved in the pathways of response to the synaptic membrane, neuron to neuron synapse, and postsynaptic density, which has been associated with depression and neural plasticity, compared to the control group BBR exhibited significant differences in genes involved in the pathways of the neuron to neuron synapse, postsynaptic specialization, synaptic membrane, and postsynaptic density compared to CORT (Fig. 3g, h). KEGG pathway enrichment analysis of the DEGs between control and CORT group found that MAPK signaling pathway, axon guidance, HIF-1 signaling pathway, and steroid biosynthesis. Both MAPK and HIF-1 are upstream signals for NLRP3 inflammasome via mediated activation of NF-κB (Fig. 3i, j). Together, these results indicate that CORT may induce neuroinflammation as well as neuroplasticity deficit in the PFC. Therefore, we hypothesized that neuronal anomalies resulting from long-term CORT-induced chronic stress exposure are associated with NLRP3 inflammasome and synaptic dysfunction in the brain networks and result in emotional episodes alteration.

### Berberine reverses neuroinflammation in the prefrontal cortex and hippocampus by inhibiting the NLRP3 signaling pathway

**Fig. 4:**
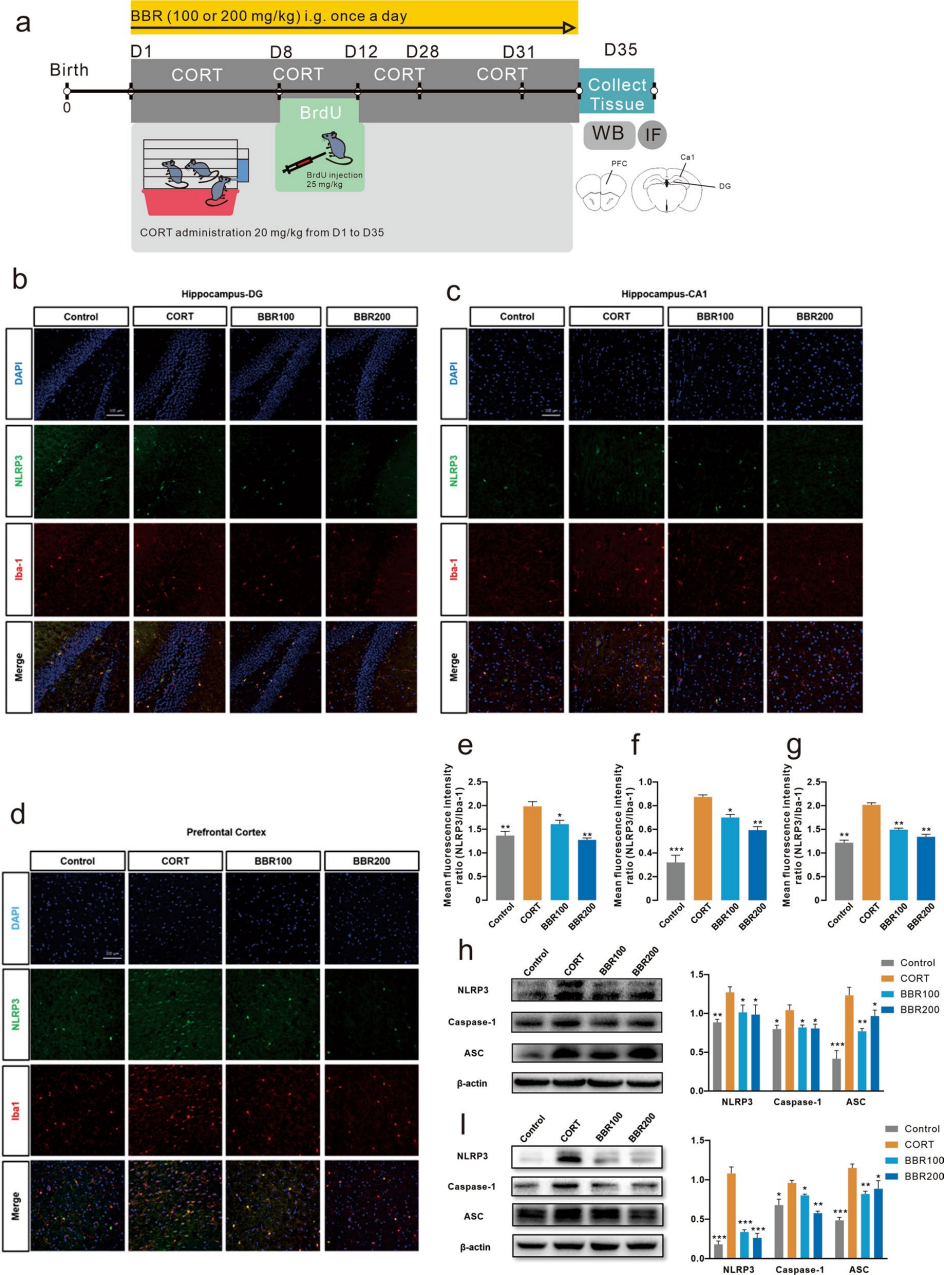
Berberine reverses neuroinflammation in the prefrontal cortex and hippocampus by inhibiting the NLRP3 inflammasome. **a** Representation of the timing of BrdU injection and illustration of an image of the target of PFC and hippocampus. **b** Immunofluorescence of NLRP3 inflammasome in the dentate gyrus subregion of the hippocampus. Scar bar, 100 µm. **c** Immunofluorescence of NLRP3 inflammasome in the CA1 subregion of the hippocampus. Scar bar, 100 µm. **d** Immunofluorescence of NLRP3 inflammasome in the PFC. Scar bar, 100 µm. **e** NLRP3 + cells (green) and Iba1 + cells (red) in DG of hippocampus, CORT increased NLRP3 + /Iba1 + cells in DG (One-way ANOVA, *F* (3, 8) = 18.63, *P* = 0.0006; Dunnett’s multiple comparisons test, CORT vs. Control, *P* = 0.0002, CORT vs. BBR100, *P* = 0.0112, CORT vs. BBR200, *P* = 0.0047, *n* = 3 in each group). **f** NLRP3 + cells (green) and Iba1 + cells (red) in CA1 of hippocampus, CORT increased NLRP3 + /Iba1 + cells in CA1 (One-way ANOVA, *F* (3, 8) = 12.28, *P* = 0.0023; Dunnett’s multiple comparisons test, CORT vs. Control, *P* = 0.0021, CORT vs. BBR100, *P* = 0.7604, CORT vs. BBR200, *P* = 0.0115, *n* = 3 in each group). **g** NLRP3 + cells (green) and Iba1 + cells (red) in PFC, CORT increased the NLRP3 + /Iba1 + cells in PFC (One-way ANOVA, *F* (3, 8) = 141.0, *P* < 0.0001; Dunnett’s multiple comparisons test, all comparisons *P* < 0.0001, *n* = 3 in each group). **h** The western blotting of NLRP3/Caspase-1/ASC in the PFC, the CORT mice showed a significant difference in the NLRP3 signaling pathway (Two-way ANOVA, row factor *F* (2, 36) = 10.30, *P* = 0.0003, column factor *F* (3, 36) = 24.85, *P* < 0.0001; Dunnett’s multiple comparisons test, all comparisons *P* < 0.0001, *n* = 3 in each group). **i** The western blotting of NLRP3/Caspase-1/ASC in the hippocampus, the CORT mice showed a significant difference in the NLRP3 signaling pathway (Two-way ANOVA, row factor *F* (2, 41) = 25.79, *P* < 0.0001, column factor *F* (3, 41) = 37.04, *P* < 0.0001; Dunnett’s multiple comparisons tests, all comparisons *P* < 0.0001, *n* = 3 in each group). Bar graphs show the mean ± SEM; *P < 0.05, **P < 0.01, ***P < 0.001. *CON* control, *CORT* corticosterone, *BBR* berberine, *ANOVA* analysis of variance, *NLRP3* NOD-like receptor thermal protein domain associated protein 3, *qPCR* quantitative reverse transcription polymerase chain reaction, *PFC* prefrontal cortex

Long-term CORT administration activates the inflammasome via NLRP3 and its linked signaling molecules, including caspase-1 and ASC regulation, which subsequently play a significant role in neuroinflammation and neurotoxicity. We examined the NLRP3 expression in PFC and hippocampus (Fig. 4a). Enhanced NLRP3 expression was detected in the hippocampal DG and CA1 subregions as well as the prefrontal cortex in CORT-induced depression mice (Fig. 4b–d). Both low and high doses of berberine treatment significantly reduced inflammasome activation, as demonstrated by decreased expression of NLRP3 (Fig. 4e–g). The western blotting results were consistent with the immunofluorescence labeling, indicating that long-term CORT administration remarkably increased the expression levels of NLRP3, caspase-1, and ASC in the hippocampus and prefrontal cortex in mice brain. Berberine treatment reversed the activation of the NLRP3 inflammasome, as demonstrated by decreased expression levels of NLRP3, caspase-1, and ASC (Fig. 4h, i). We further investigated whether the level of NLRP3 inflammasome could be correlated with key behavioral features of mice including time in center zone of OFC, number of zone transition of OFT, immobility duration of FST, immobility duration of TST, and sucrose preference of SPT. The NLRP3 inflammasome data obtained from 28 mice (*n* = 7 in each group) were pooled for correlation analysis. The components of NLRP3 inflammasome showed highly correlation between each other. A cluster of prefrontal NLRP3 inflammasome level inversely correlated with time in central zone, zone transition number, and sucrose preference. However, the NLRP3 inflammasome level in the PFC showed a positive correlation with immobile duration (Additional file 1: Fig. S3). These findings are consistent with the overall conclusions from the results presented in mice, indicating that NLRP3 expression is elevated in depressed mice and is decreased in the normal. Together, these results identify that CORT induced depression of mice via an elevated NLRP3 signaling pathway, while berberine exhibited antidepressant effects via a suppressed NLRP3 signaling pathway.

### Effects of berberine on neural plasticity and hippocampal adult neurogenesis

**Fig. 5:**
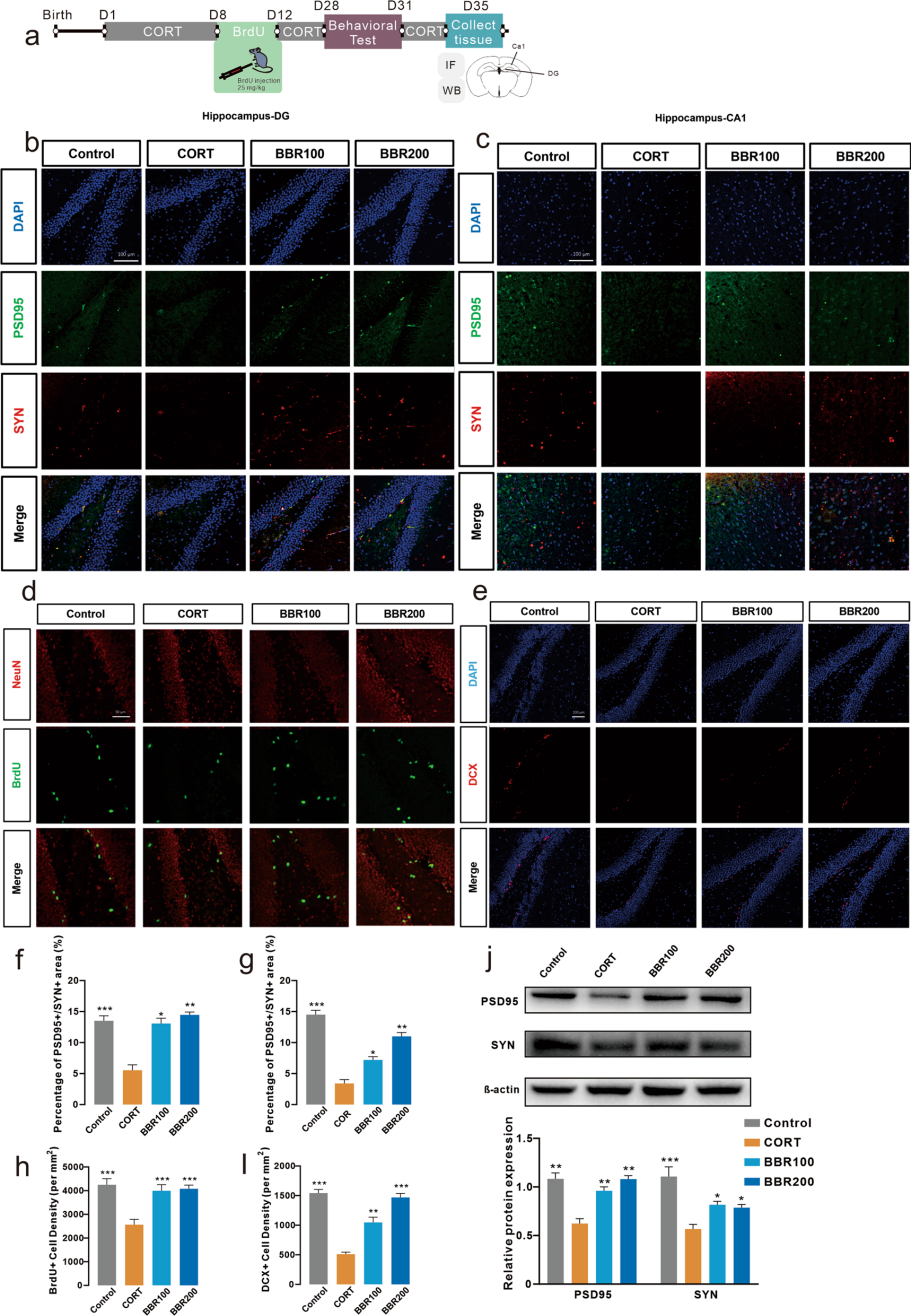
Berberine increases hippocampal DG and CA1 subregional synaptic plasticity and adult neurogenesis induced by CORT. **a** Representation of the timing of experiments and illustration of the image of the target of PFC and hippocampus. **b** Hippocampal DG images show the decreasing signal intensity of PSD95 and SYN in the CORT group, and both BBR100 and BBR200 increased the PSD95 + and SYN + cell number (One-way ANOVA, *F* (3, 8) = 10.12, *P* = 0.0043; Dunnett’s multiple comparisons test, CORT vs. Control, *P* = 0.0021, CORT vs. BBR100, *P* = 0.0251, CORT vs. BBR200, *P* = 0.0081, *n* = 3 in each group). Scar bar, 100 µm. **c** Representation of the immunofluorescence labeling of hippocampal CA1, CORT significantly decreased the PSD95 + and SYN + cell number compared to BBR100 and BBR200 (One-way ANOVA, *F* (3, 8) = 84.63, *P* < 0.0001; Dunnett’s multiple comparisons test, all comparisons *P* < 0.0001, *n* = 3 in each group). Scar bar, 100 µm. **d** NeuN (red) and BrdU (green) in hippocampal DG images (One-way ANOVA, *F* (3, 8) = 155.0, *P* < 0.0001; Dunnett’s multiple comparisons test, all comparisons *P* < 0.0001, *n* = 3 in each group). Scar bar, 50 µm. **e** DAPI (blue) and DCX (red) in hippocampal DG images (One-way ANOVA, *F* (3, 8) = 220.0, *P* < 0.0001; Dunnett’s multiple comparisons test, all comparisons *P* < 0.0001, *n* = 3 in each group). Scar bar, 100 µm. **f** percentage of PSD95 + /SYN + area in hippocampal DG. **g** percentage of PSD95 + /SYN + area in hippocampal CA1. **h** BrdU + cell density in hippocampal DG. **i** DCX + cell density in hippocampal DG. **j** Western blotting assay for PSD95 and SYN, the CORT mice showed a significant difference in the expression of PSD95 and SYN (Two-way ANOVA, row factor *F* (1, 19) = 11.90, *P* = 0.0027, column factor *F* (3, 19) = 30.06, *P* < 0.0001; Dunnett’s multiple comparisons test, all comparisons *P* < 0.0001, *n* = 3 in each group). Bar graphs show the mean ± SEM; *P < 0.05, **P < 0.01, and ***P < 0.001. *CORT* corticosterone, *BBR* berberine, *ANOVA* analysis of variance, *DG* dentate gyrus, *DCX* Doublecortin, *BrdU* Bromodeoxyuridine, *PSD95* postsynaptic density protein 95, *SYN* synapsin

Combined with the alterations of behaviors and cytokine levels, we hypothesize that long-term administration of CORT induced neurotoxicity via neuroinflammation that is associated with NLRP3 inflammasome, resulting in cognitive deficits, which disrupts synaptic plasticity and contributes to dysregulated synaptogenesis. To further explore whether the effects of berberine could regulate neuroplasticity and neurogenesis, we detect the spine density in synaptic plasticity and neurogenesis in the hippocampus of mice via western blotting, immunofluorescence labeling, Golgi-Cox staining, and TEM (Fig. 5a). In the hippocampal DG and CA1 subregions of CORT mice, the expression levels of PSD95 and SYN were significantly decreased (Fig. 5b, c, f, g). Both low and high doses of berberine treatment significantly attenuated CORT-induced changes, indicating that berberine reduced the synaptic defects generated under CORT-induced stress results (Fig. 5b, c, f, g). The immunofluorescence labeling illustrated that the long-term CORT administration resulted in a significant reduction density of newly generated immature neuron labels with BrdU and DCX in the hippocampus in mice (Fig. 5d, e, h, i). Berberine treatment also increased both BrdU and DCX positive cells (Fig. 5d, e, h, i).

**Fig. 6:**
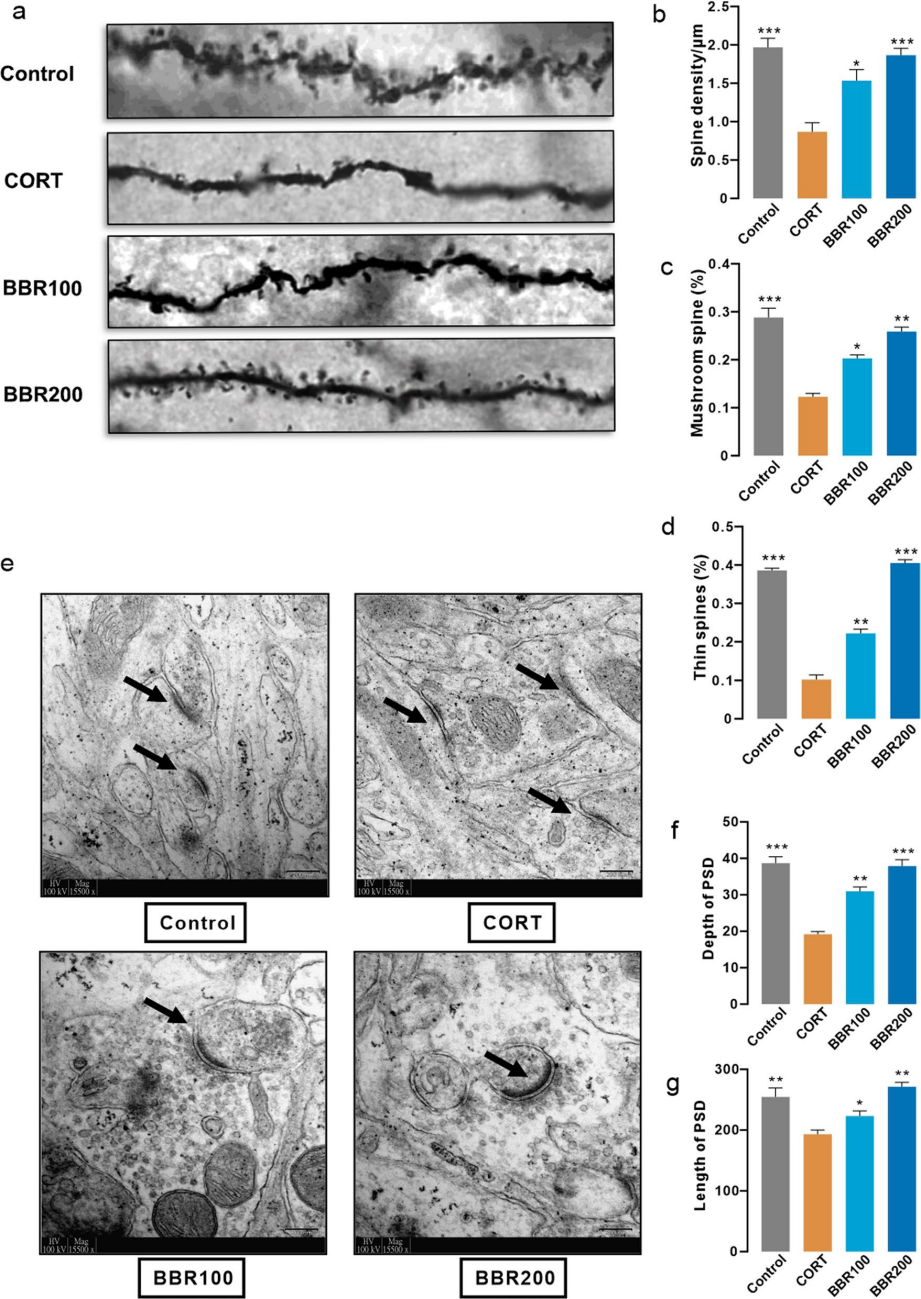
Berberine increases hippocampal adult neurogenesis induced by CORT.** a** Representation of the Golgi-Cox staining of hippocampal adult neuron. **b** Spine density results from Golgi staining (One-way ANOVA, *F* (3, 8) = 17.10, *P* = 0.0008; Dunnett’s multple comparisons test, CORT vs. Control, *P* = 0.0005, CORT vs. BBR100, *P* = 0.0112, CORT vs. BBR200, *P* = 0.0010, *n* = 3 in each group). **c** Proportion of mushroom spines (One-way ANOVA, *F* (3, 8) = 6.068, *P* < 0.0001; Dunnett’s multple comparisons test, CORT vs. Control, *P* = 0.0117, CORT vs. BBR200, *P* = 0.0432, *n* = 3 in each group). **d** Proportion of thin spines (One-way ANOVA, *F* (3, 8) = 52.74, *P* < 0.0001; Dunnett’s multple comparisons test, CORT vs. Control, *P* = 0.0001, CORT vs. BBR100, *P* = 0.0054, CORT vs. BBR200, *P* < 0.0001, *n* = 3 in each group). **e** Representative electron micrographs showing the synaptic structure and postsynaptic densities on neurons. Scar bar, 200 µm. **f** Depth of post synaptic dendrites (One-way ANOVA, *F* (3, 8) = 38.93, *P* < 0.0001; Dunnett’s multple comparisons test, CORT vs. Control, *P* < 0.0001, CORT vs. BBR100, *P* = 0.0033, CORT vs. BBR200, *P* = 0.0001, *n* = 3 in each group). **g** Length of post synaptic dendrites (One-way ANOVA, *F* (3, 8) = 220.0, *P* < 0.0001; Dunnett’s multple comparisons test, all comparisons *P* < 0.0001, *n* = 3 in each group). Bar graphs show the mean ± SEM; *P < 0.05, **P < 0.01, and ***P < 0.001. *CORT* corticosterone, *BBR* berberine, *ANOVA* analysis of variance

To confirm the function of dendritic function and morphology, the elimination of postsynaptic dendritic spines on the hippocampus was observed by Golgi-Cox staining. The results showed a significant loss of synaptic density in the mice after long-term CORT exposure (Fig. 6a, b). To explore how CORT exposure is related to dendritic spines, we classified dendritic spines into two categories based on the maximal diameter of the spine head (both thin and mushroom spines maximal diameter < 0.6 µm) and the length of spines (thin spines maximal length > 0.9 µm vs. mushroom spines maximal length < 0.9 µm). Long-term CORT administration markedly decreased the number of both mushroom and thin dendrite spines (Fig. 6a, c, d). Both low dosage (100 mg/kg/day) and high dosage (200 mg/kg/day) of berberine treatment attenuated the decrease of several spines in CORT-treated mice (Fig. 6a, c, d). The results from TEM also suggested that CORT-induced stress condition significantly decreases both mean depth and length of postsynaptic densities in CORT-induced mice than in control and berberine treatment groups (Fig. 6e–g).

### The effects of berberine on long-term CORT administration impaired the synaptic function of pyramidal neurons in the mPFC

**Fig. 7:**
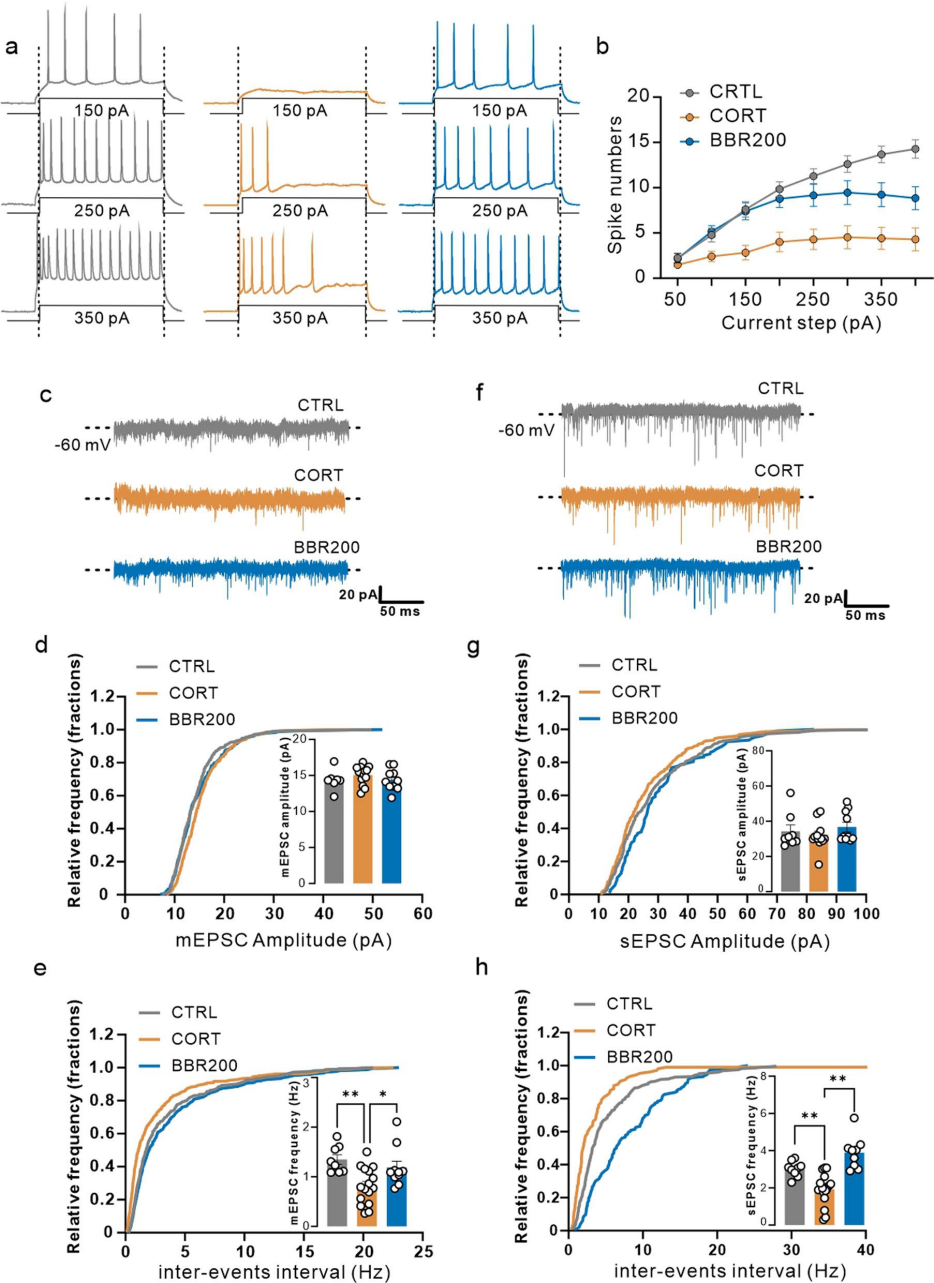
Berberine prevents CORT-induced patch-clamp alteration. **a** Representation of the data from the excitatory neuron of contrl (black), CORT (red), and BBR 200 mg/kg/day (green), which were stimulated by electricity from 50 to 350 pA, **b** representation of the data from the excitatory neuron, which were stimulated by electricity from 50 to 400 pA and records of the number of spikes (Two-way ANOVA, row factor *F* (7, 360) = 22.10, *P* < 0.0001, column factor *F* (2, 360) = 86.48, *P* < 0.0001; Tukey’s multple comparisons test, for 150 pA, CORT vs. control, *P* = 0.0008, CORT vs. BBR, *P* = 0.0042; for 250 pA, CORT vs. control, *P* < 0.0001, CORT vs. BBR, *P* = 0.0021; for 350 pA, CORT vs. control, *P* < 0.0001, CORT vs. BBR, *P* = 0.0023, *n* = 3 in each group). **c** Representation of the mEPSC of contrl (black), CORT (red), and BBR 200 mg/kg/day (green) **d** Representation of the amplitude of mEPSC (One-way ANOVA, *F* (2, 31) = 1.038, *P* = 0.3662). **e** Frequency of mEPSC was significantly lower in CORT compared to CON and BBR 200 mg/kg/day (One-way ANOVA, *F* (2, 31) = 6.972, *P* = 0.0032; Tukey’s multple comparisons test, CORT vs. control, *P* = 0.0046, CORT vs. BBR, *P* = 0.0388, *n* = 3 in each group). **f** Representation of the sEPSC of contrl (black), CORT (red), and BBR 200 mg/kg/day (green) **g** Representation of the amplitude of sEPSC (One-way ANOVA, *F* (2, 30) = 0.7402, *P* = 0.4855, *n* = 3 in each group). **h** Representation of the the frequency of sEPSC was significantly lower in CORT compared to CON and BBR 200 mg/kg/day (One-way ANOVA, *F* (2, 32) = 20.70, *P* < 0.0001; Tukey’s multple comparisons test, CORT vs. control, *P* = 0.0040, CORT vs. BBR, *P* < 0.0001, *n* = 3 in each group). Bar graphs show the mean ± SEM; *P < 0.05, **P < 0.01, and ***P < 0.001. CORT, corticosterone; BBR, berberine; ANOVA, analysis of variance; mEPSC, miniature excitatory postsynaptic current; sEPSC, spontaneous excitatory postsynaptic current

NLRP3 inflammasome activation induced neuroinflammatory response may also negatively affect the neuroplasticity, and the GO and KEGG analysis from RNA-seq results suggested that long-term CORT exposure involved the function of the synapse. To detect whether long-term CORT exposure alters the excitability of mPFC, the frequency of action potentials in response to depolarizing current steps was measured from pyramidal neurons in the mPFC. The number of action potentials elicited (induced spikes) throughout 500 ms was measured, as the current was varied in steps of 50 pA from − 50 to 400 pA (Fig. 7a, b). The pyramidal neurons were characterized as the cells that displayed spike-frequency adaption and broad action potentials and lacked spontaneous discharge at resting membrane potential. Depolarizing currents evoked higher firing in the mice of the control group and berberine-treated (BBR200) mice than in the CORT group. Similarly, less current was required to drive the cell to fire spikes at a given frequency (Fig. 7a, b). To examine the functional consequences of long-term CORT exposure on the synaptic transmission in the mPFC, a whole-cell patch-clamp recording of pyramidal neurons was performed. Spontaneous excitatory postsynaptic currents (sEPSCs) and mini excitatory postsynaptic currents (mEPSCs) were recorded in the same cells by alternate clamping at the reversal potential of glutamate receptor-mediated and α-amino-3-hydroxy-5-453 methylisoxazole-4-propionic acid receptor (AMPAR)-mediated currents, respectively (Fig. 7c, f). The sEPSCs and mEPSCs amplitude were comparable among groups (Fig. 7d, g), while for frequency of sEPSCs and mEPSCs, whereas CORT administration significantly inhibited both the frequency of sEPSCs and mEPSCs compared with mice in control and berberine group (200 mg/kg) (Fig. 7e, h), suggesting a deficit in CORT inhibits synaptic transmission and abnormal discharge in mPFC pyramidal neurons, which may then contribute to the depression-like behaviors observed in CORT administration mice.

## Discussion

Despite the growing number of evidence suggesting a strong link between neuropsychiatric disorders and immune responses. To date, pharmacological therapies with satisfactory effectiveness are still elusive [19]. The current findings indicate that after long-term CORT exposure, the induced depression-like behaviors were associated with remarkably neuroinflammatory responses regarding NLRP3 inflammasome and cytokines as well as electrophysiological function in PFC and structural neuronal in the hippocampus of mice. Notably, berberine attenuated the behavioral alterations and regulated the NLRP3 signaling pathway with overexpressed cytokines, consequently, reversed the deterioration of neural plasticity induced by CORT.

In this study, DEG analysis were initially identified in PFC via assessing the functions of genes by using high-throughput sequencing. NLRP3 was found to be overexpressed as a hub gene in the PFC of CORT-induced depression mice, suggesting that NLRP3 might play a key role and take part in the development and pathogenesis of depression. Findings from previous preclinical and clinical studies have indicated that NLRP3 inflammasome-driven pathways might be involved in numerous neuropsychiatric disorders including neuroinflammation-induced depression [14, 27–30]. As revealed by western blotting and immunofluorescence labeling, the current data suggest that the NLRP3 signaling pathway was activated in CORT-induced depression mice, while berberine downregulated the NLRP3 signaling pathway in the hippocampus and PFC. IL-1β is one of the main mediators of the crosstalk between the immune system and the CNS. The NLRP3 inflammasome processes pro-IL-1β into mature interleukins and act as a pro-inflammatory mediator, it has been confirmed that IL-1β was elevated in the serum of depression patients and associated with depression inventory scores [31, 32]. Together, these results confirmed that NLRP3 was involved in the process of neuroinflammation and served as a modulator in the development of depression. Meanwhile, several studies have demonstrated that depression is accompanied by dendritic remodeling in neurons of the PFC and hippocampal [33–35]. The brain-derived RNA could regulate dendritic spine development and may thus be involved in regulating neural plasticity and behaviors [36]. The structural and morphologic characteristics of neurons from Golgi-staining and TEM showed that CORT induced specific alterations in the synapse. In addition, CORT administration decreased excitatory synaptic transmission function as revealed from electrophysiological recordings of mEPSCs and sEPSCs in pyramidal neurons in the mPFC. These changes might be attributed to impaired neurobiological functions as associated with the NLRP3 inflammasome activation.

It is well-documented that glucocorticoids are efficacy anti-inflammatories and have been frequently applied for inflammatory conditions including autoimmune diseases. However, hypersecretion glucocorticoids lay a foundation for the neuroinflammation hypothesis regarding the pathogenesis of depression [37, 38]. Immunosuppressive activities of glucocorticoids negatively regulate pro-inflammatory related pathways including glucocorticoid-mediated activation [39]. Specifically, Toll-like receptors (TLRs) could be activated by glucocorticoids, increasing the expression of several members of the TLRs family which is critical for inflammatory response [40]. As the agonist of the glucocorticoid receptor, CORT activated glucocorticoid receptor increased the expression of purinergic receptor and result in the over-secretion of IL-6 [41, 42]. Meanwhile, it can stimulate the activation of the NLRP3 inflammasome, by facilitating NLRP3 induction and inflammasome formation, glucocorticoids promote activation of neuroinflammation and the release of pro-inflammatory cytokine IL-1β. TLRs in circulating monocytes could be activated by DAMPs or PAMPs, the NLRP3 inflammasome transcription and combine ASC and pro-caspase-1, then processes pro-IL-1β to IL-1β, leading to release of mature cytokines in the extracellular milieu and inflammatory response [15]. The overproduction of cytokines such as IL-1β, TNF-α, and IFN-γ further result in the dysfunction of monoamine metabolism, including overexpression of indoleamine 2,3-dioxygenase (IDO), leading to the production of kynurenine metabolites from tryptophan [43]. As a natural IDO inhibitor, berberine decreased the production of kynurenine, which is subsequently converted into metabolites having modulatory effects on glutamatergic neurotransmission [44, 45]. These findings illustrate that the potential anti-depressant effects of berberine are attributed to its anti-inflammatory especially for inhibiting NLRP3 inflammasome activation. It should be noted that our results were limited to PFC and hippocampus, some other brain regions related to depression such as hypothalamus, amygdala, and locus coeruleus were not involved in the current study.

## Conclusion

In conclusion, the findings of this study illustrated that berberine, via suppression of the NLRP3/caspase-1/ASC/IL-1β signaling pathway, play a key role in the alleviation of neuronal anomalies accompanied by emotional alteration induced by long-term CORT administration. Berberine attenuates the impairment of neuroplasticity and neurogenesis by inhibiting the neuroinflammatory response in PFC and hippocampus.

## Supplementary Information

**Additional file 1.** Library preparation, transcriptome sequencing, and bioinformatic analysis for RNA-seq data. **Fig. S1.** Body weight of mice during experiments. **Fig. S2.** Uncropped western blot imaging. **Fig. S3.** Scatter plots of linear regression demonstrating the association between behavioral parameters and NLRP3 inflammasome. **Table S1.** The primers used in qPCR. **Table S2.** Antibody information for Western blotting. **Table S3.** Antibody information of immunofluorescence. **Table S4.** Sequencing quality control measures.

## Data Availability

The raw data supporting the conclusions of this article will be made available by the authors, without undue reservation, to any qualified researcher upon request.

## Abbreviations

ANOVA: Analysis of variance
BBB: Blood–brain barrier
BBR: Berberine
CCMR: Centre for Comparative Medicine Research
CNS: Central nervous system
CORT: Corticosterone
CULATR: Committee on the Use of Live Animals in Teaching and Research
DALYs: Disability-adjusted life-years
DAMPS: Danger-associated molecular patterns
DEG: Differentially expressed gene
ELISA: Enzyme-linked immunosorbent assay
FST: Forced swim test
HPA: Hypothalamus–pituitary–adrenal
NLRP3: NOD-like receptor thermal protein domain associated protein 3
OFT: Open field test
PAMPS: Pathogen-associated molecular patterns
PFC: Prefrontal cortex
qPCR: Quantitative reverse transcription polymerase chain reaction
SPT: Sucrose preference test
TEM: Transmission electron microscope
TST: Tail suspension test
