# Supplementary material for: Berberine ameliorates depression-like behaviors in mice via inhibiting NLRP3 inflammasome-mediated neuroinflammation and preventing neuroplasticity disruption
Source: J Neuroinflammation. 2023 Mar 1;20:54. doi: 10.1186/s12974-023-02744-7 (PMC9976521; doi:10.1186/s12974-023-02744-7)
Supplement: Supplementary file 1 — Additional file 1. Library preparation, transcriptome sequencing, and bioinformatic analysis for RNA-seq data. Fig. S1. Body weight of mice during experiments. Fig. S2. Uncropped western blot imaging. Fig. S3. Scatter plots of linear regression demonstrating the association between behavioral parameters and NLRP3 inflammasome. Table S1. The primers used in qPCR. Table S2. Antibody information for Western blotting. Table S3. Antibody information of immunofluorescence. Table S4. Sequencing quality control measures. [file 12974_2023_2744_MOESM1_ESM.docx]

Additional material

Berberine ameliorates depression-like behaviors in mice via inhibiting NLRP3 inflammasome mediated neuroinflammation and preventing neuroplasticity disruption

Zongshi Qin ^a, 1^, Dong-Dong Shi ^b, 1^, Wenqi Li ^a^, Dan Cheng ^a^, Ying-Dan Zhang ^b^, Sen Zhang ^b^, Bun Tsoi ^c^, Jia Zhao ^a^, Zhen Wang ^b,*^, Zhang-Jin Zhang ^a,*^

*^a^ School of Chinese Medicine, LKS Faculty of Medicine, The University of Hong Kong, Hong Kong, China*

*^b^ Shanghai Mental Health Center, Shanghai Jiao Tong University School of Medicine, Shanghai, China*

*^c^ Department of Applied Biology and Chemical Technology, The Hong Kong Polytechnic University, Hong Kong Special Administrative Region, China*

^1^ These authors equally contributed to this study.

^*^ Corresponding author: Zhen Wang, Shanghai Mental Health Center, Shanghai Jiao Tong University School of Medicine, Shanghai, China. E-mail: wangzhen@smhc.org.cn; Zhang-Jin Zhang, School of Chinese Medicine, LKS Faculty of Medicine, The University of Hong Kong, Hong Kong, China. E-mail: zhangzj@hku.hk

Materials and methods

Library preparation, transcriptome sequencing, and bioinformatic analysis for RNA-seq data

RNA integrity was assessed using the RNA Nano 6000 Assay Kit of the Bioanalyzer system (2100, Agilent Technologies, USA). Total RNA was used as input material for the RNA sample preparation. Briefly, mRNA was purified from total RNA using poly-T oligo-attached magnetic beads. Fragmentation was carried out using divalent cations under elevated temperature in First Strand Synthesis Reaction Buffer. First-strand cDNA was synthesized using random hexamer primer and M-MuLV Reverse Transcriptase. Second strand cDNA synthesis was subsequently performed using DNA Polymerase I and RNase H. Remaining overhangs were converted into blunt ends via exonuclease/polymerase activities. After adenylation of 3’ends of DNA fragments, an adaptor with a hairpin loop structure was ligated to prepare for hybridization. To select cDNA fragments of preferentially 370~420 bp in length, the library fragments were purified with the AMPure XP system (Beckman Coulter, USA) and the PCR was performed with Phusion High-Fidelity DNA polymerase, Universal PCR primers, and Index (X) Primer. Finally, PCR products were purified (AMPure XP system) and library quality was assessed on the Agilent Bioanalyzer system (2100, Agilent Technologies, USA). Raw data (raw reads) of fastq format were initially processed through in-house Perl scripts. Briefly, clean reads were obtained after removing reads containing adapter and poly-N and reads with low quality. Meanwhile, Q20, Q30, and GC content the clean reads were calculated. All the downstream analyses were based on high-quality clean data. Reference genome and gene model annotation files were downloaded from NCBI Datasets Genome Data Package. Index of the reference genome was built using Hisat2 v2.0.5 and paired-end clean reads were aligned to the reference genome using Hisat2 v2.0.5. Hisat2 was selected as the mapping tool which can generate a database of splice junctions based on the gene model annotation file and thus a better mapping result than other non-splice mapping tools. The mapped reads of each sample were assembled by StringTie (v1.3.3b) in a reference-based approach, which used a novel network flow algorithm as well as an optional de novo assembly step to assemble and quantitate full-length transcripts representing multiple splice variants for each gene locus. FeatureCounts (v1.5.0) was used to count the reads numbers mapped to each gene. The Fragments Per Kilobase of exon model per Million mapped fragments (FPKM) of each gene was calculated based on the length of the gene and read count mapped to this gene.

R (v.4.1.1) was used for differentially expressed gene analysis and visualization of RNA-seq data. Specifically, differential expression analysis of two groups was performed using the DESeq2 package (v1.20.0) of R, which provided statistical routines for determining differential expression in digital gene expression data using a model based on the negative binomial distribution. The resulting P-values were adjusted using the Benjamini & Hochberg’s approach for controlling the false discovery rate. Genes with an adjusted P-value less than 0.05 found by DESeq2 were assigned as differentially expressed. Before differential gene expression analysis, for each sequenced library, the read counts were adjusted by the edgeR program package through one scaling normalized factor. Differential expression analysis of two conditions was performed using the edge R (v3.22.5). The P-value was adjusted using the Benjamini & Hochberg method. Corrected P-value of 0.05 and absolute foldchange of 2 was set as the threshold for significantly differential expression. Gene Ontology enrichment analysis of differentially expressed genes were implemented by the clusterProfiler R package (v3.15), in which gene length bias was corrected. GO terms with corrected P-value less than 0.05 were considered significantly enriched by differential expressed genes. KEGG is a database resource for understanding high-level functions and utilities of the biological system, such as the cell, the organism and the ecosystem, from molecular-level information, especially large-scale molecular datasets generated by genome sequencing and other high-through put experimental technologies. clusterProfiler R package was used to test the statistical enrichment of differential expression genes in KEGG pathways.

Fig. S1. Body weight of mice during experiments

The CORT administration significantly decreased body weight in mice compared to the control group. From day 21 to day 35, high-dose berberine (200 mg/kg) treatment reversed the body weight and from day 28 to day 35, low-dose berberine (100 mg/kg) treatment showed similar effects regarding body weight changes (Two-way ANOVA, Row factor F (5, 231) = 43.96, P<0.0001, Column factor F (3, 231) = 20.78, P<0.0001).


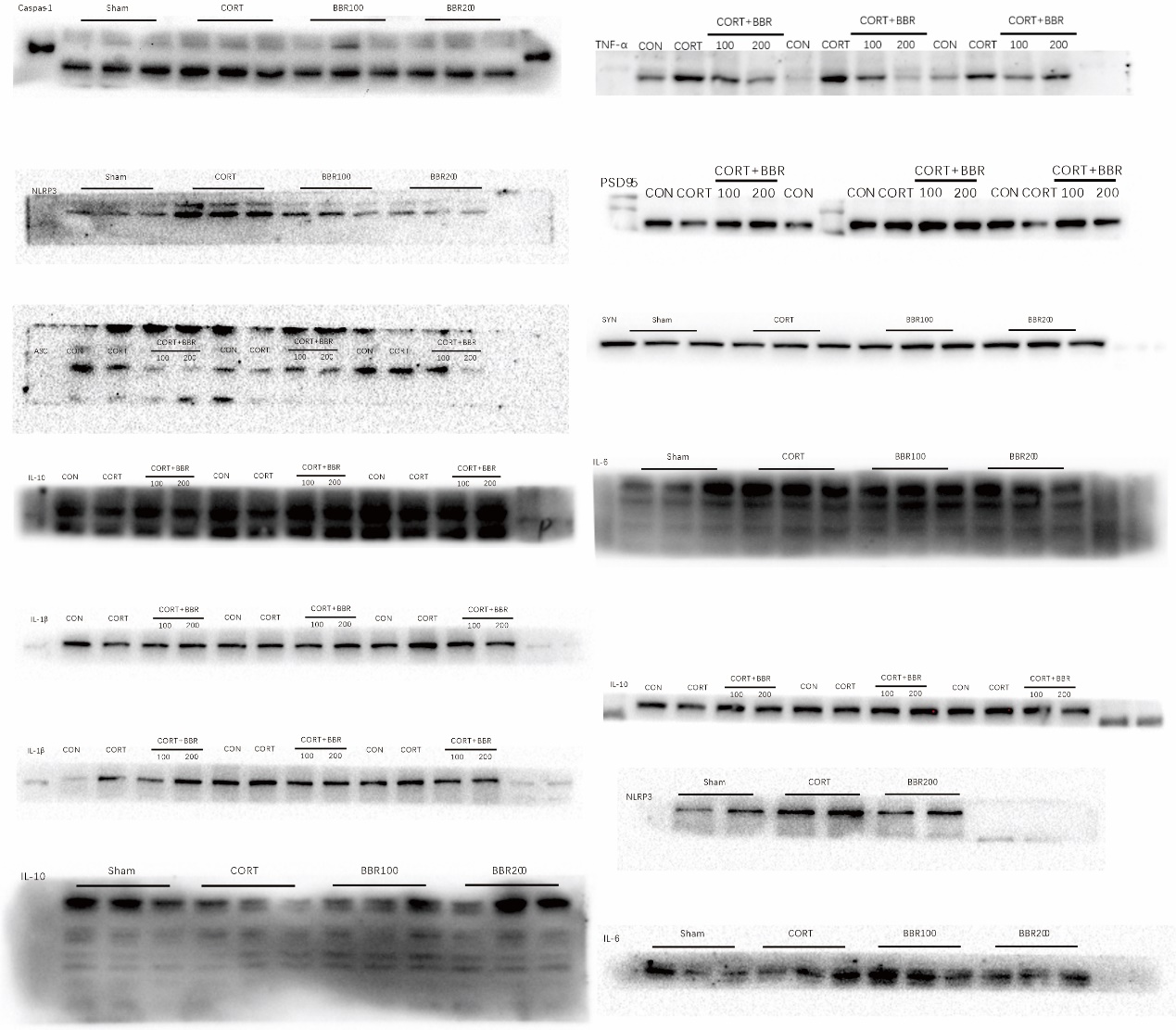


Fig. S2. Uncropped Western blot imaging.


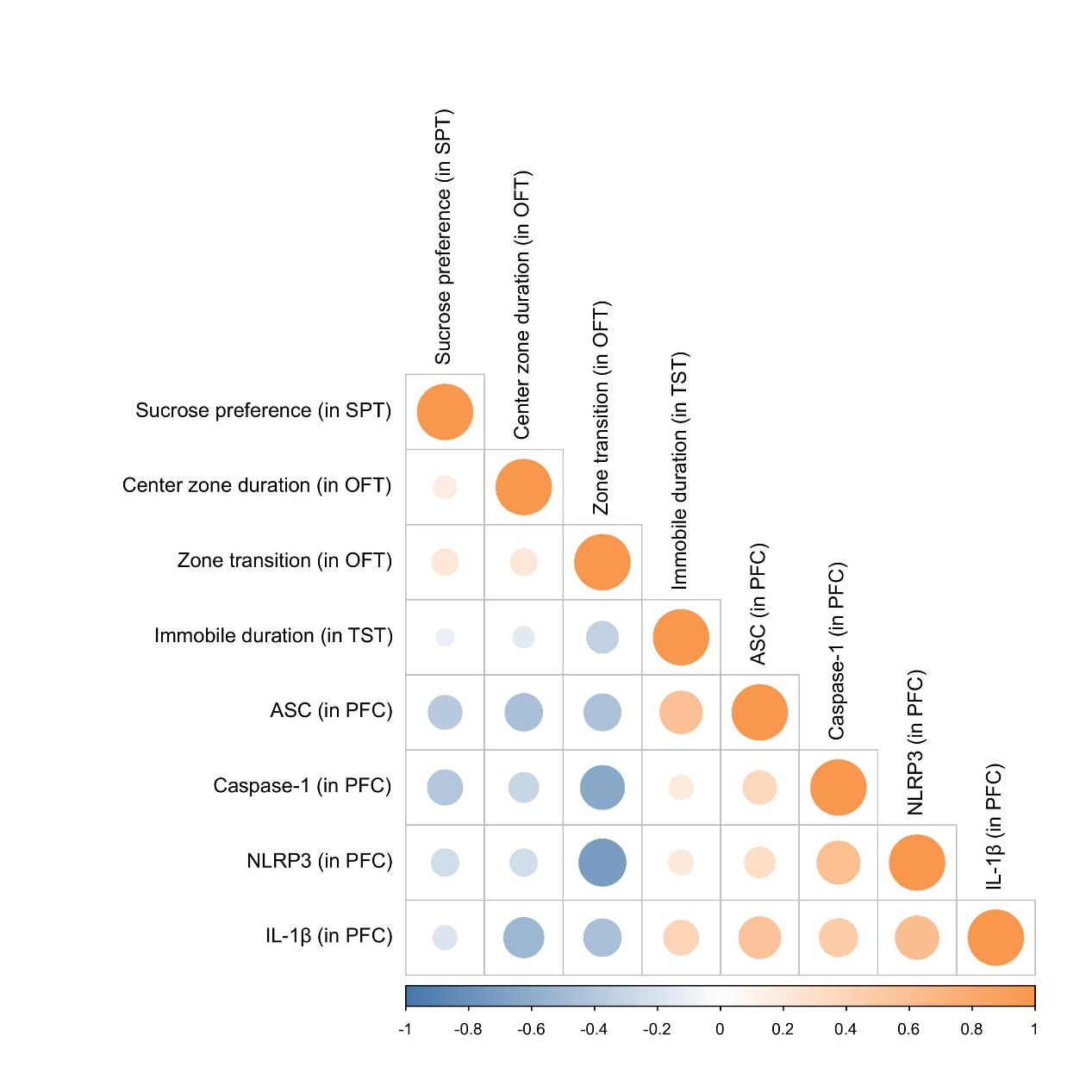


Fig. S3. Associations of the behavioral features in mice and NLRP3 inflammasome level

The Spearman’s correlation coefficient was calculated. Orange bubbles denotes negative relationship and blue denotes positive relationship. The bubble size is related to p-values, a larger size bubble has a less p value.

Table S1. The primers used in qPCR

| Primer | Sequence |
| --- | --- |
| NLRP3 forward | 5′-GAGCTGGACCTCAGTGACAATGC-3′ |
| NLRP3 reverse | 5′-ACCAATGCGAGATCCTGACAACAC-3′ |
| Caspase1 forward | 5′-GAGCTGATGTTGACCTCAGAG-3′ |
| Caspase1 reverse | 5′-CTGTCAGAAGTCTTGTGCTCTG-3′ |
| IL-1beta forward | 5′-CAACTGTTCCTGAACTCAACTG-3′ |
| IL-1beta reverse | 5′-GAAGGAAAAGAAGGTGCTCATG-3′ |
| β-actin forward | 5′-GGACTTCGAGCAAGAGATGG-3′ |
| β-actin reverse | 5′-AGCACTGTGTTGGCGTACAG-3′ |

Table S2. Antibody information for Western blotting

| Antibody | Manufacture | Dilution | Host |
| --- | --- | --- | --- |
| IL-6 | Abcam | 1:1000 | Mouse |
| IL-1β | Proteintech | 1:1000 | Rabbit |
| Pro-1L-1β | Abcam | 1:1000 | Rabbit |
| IL-10 | Proteintech | 1:1000 | Rabbit |
| TNF-α | Proteintech | 1:1000 | Rabbit |
| NLRP3 | Abcam | 1:1000 | Gout |
| Caspase-1 | Proteintech | 1:1000 | Rabbit |
| ASC | Proteintech | 1:1000 | Rabbit |
| PSD95 | Abcam | 1:1000 | Rabbit |
| SYNAPHY | Proteintech | 1:1000 | Mouse |
| β-actin | Proteintech | 1:1000 | Mouse |

Table S3. Antibody information of immunofluorescence

| Antibody | Manufacture | Dilution | Host |
| --- | --- | --- | --- |
| BrdU | Proteintech | 1:200 | Mouse |
| NeuN | Abcam | 1:200 | Rabbit |
| NLRP3 | Proteintech | 1:100 | Rabbit |
| Iba-1 | Abcam | 1:100 | Goat |
| DCX | ABclonal | 1:500 | Rabbit |
| PSD95 | Abcam | 1:100 | Rabbit |
| SYNAPSIN | Proteintech | 1:100 | Mouse |

Table S4. Sequencing quality control measures

| Sample | Clean Reads No. | Clean Data (bp) | Clean Reads |
| --- | --- | --- | --- |
| CON_1 | 41114782 | 6167217300 | 89.06% |
| CON_2 | 36904224 | 5535633600 | 90.79% |
| CON_3 | 36506604 | 5475990600 | 90.01% |
| MOD_1 | 36287764 | 5443164600 | 90.12% |
| MOD_2 | 34799528 | 5219929200 | 89.63% |
| MOD_3 | 36030700 | 5404605000 | 89.07% |
| BBR_1 | 34695508 | 5204326200 | 88.68% |
| BBR_2 | 36651444 | 5497716600 | 89.71% |
| BBR_3 | 34997938 | 5249690700 | 88.82% |
